# Study of bronchial hyper-reactivity in patients with allergic rhinitis in an urban indian setup

**DOI:** 10.1186/1939-4551-8-S1-A245

**Published:** 2015-04-08

**Authors:** Priti Meshram, Dattatraya Birajdar, Nagsen Ramraje

**Affiliations:** 1 Grant Government Medical College, India

## Background

The concept of Atopic march was developed to study the progress of Atopic Dermatitis to Allergic Rhinitis to Asthma. Epidemiologic studies have constantly demonstrated strong associations between Rhinitis and Asthma. Bronchial hyper-reactivity is an important component in the definition of Asthma. Many patients with Allergic Rhinitis have lower Airway hyperreactivity or bronchial hyper-responsiveness without obstructive ventilatory defect on PFT indicating that such patients may have a risk of developing Asthma. Most of these studies have been conducted in the Western countries. Our study aimed to detect the presence of Bronchial hyper-reactivity in patients with Allergic Rhinitis with normal lung function tests in our set up.

## Methods

In our study, 40 patients of Allergic Rhinitis with normal PFT were screened by Bronchial Challenge Tests (BCT) for the presence of Bronchial hyperreactivity after obtaining required ethical clearance from the Institutional Ethics Committee.

## Observations

Allergic Rhinitis, especially without any respiratory presentation was commonly seen in the younger age group. The study population comprised of predominantly male patients. Majority of the patients in the study had Mild Allergic Rhinitis. More than half the study population had Bronchial Hyper-reactivity. Of these, majority had severe Bronchial Hyper-reactivity. It was found that as the duration of symptoms increased in years, the concentration of Histamine at which the BCT was positive decreased and the correlation was found to be significant. No significant correlation was found between Allergic Rhinitis and Eosinophilia. Also, no significant correlation was found between Bronchial Hyperreactivity and Eosinophilia. A significant positive correlation was found between family history of Allergic Rhinitis and Asthma and BCT. Our study showed a significant positive correlation with Atopy and BCT.

## Conclusions

Allergic Rhinitis patients with Atopy are likely to be BCT positive and as a result likely to develop Asthma in the future. As the duration of Allergic rhinitis symptoms increased in years, the concentration of Histamine at which the BCT was positive decreased. Bronchial Challenge tests can be used as a screening tool in patients with Allergic Rhinitis to know the likelihood of developing asthma.

